# Are Virtual Rehabilitation Technologies Feasible Models to Scale an Evidence-Based Fall Prevention Program? A Pilot Study Using the Kinect Camera

**DOI:** 10.2196/rehab.4776

**Published:** 2015-11-05

**Authors:** Tiffany E Shubert, Jeanna Basnett, Anang Chokshi, Mark Barrett, Ravi Komatireddy

**Affiliations:** ^1^Shubert ConsultingChapel Hill, NCUnited States; ^2^Reflexion HealthSan Diego, CAUnited States

**Keywords:** aging, fall prevention, technology, evidence-based, Kinect, falls

## Abstract

**Background:**

Falls in older adults are a significant public health issue. Interventions have been developed and proven effective to reduce falls in older adults, but these programs typically last several months and can be resource intensive. Virtual rehabilitation technologies may offer a solution to bring these programs to scale. Off-the-shelf and custom exergames have demonstrated to be a feasible adjunct to rehabilitation with older adults. However, it is not known if older adults will be able or willing to use a virtual rehabilitation technology to participate in an evidence-based fall prevention program. To have the greatest impact, virtual rehabilitation technologies need to be acceptable to older adults from different backgrounds and level of fall risk. If these technologies prove to be a feasible option, they offer a new distribution channel to disseminate fall prevention programs.

**Objective:**

Stand Tall (ST) is a virtual translation of the Otago Exercise Program (OEP), an evidence-based fall prevention program. Stand Tall was developed using the Virtual Exercise Rehabilitation Assistant (VERA) software, which uses a Kinect camera and a laptop to deliver physical therapy exercise programs. Our purpose in this pilot study was to explore if ST could be a feasible platform to deliver the OEP to older adults from a variety of fall risk levels, education backgrounds, and self-described level of computer expertise.

**Methods:**

Adults age 60 and over were recruited to participate in a one-time usability study. The study included orientation to the program, navigation to exercises, and completion of a series of strength and balance exercises. Quantitative analysis described participants and the user experience.

**Results:**

A diverse group of individuals participated in the study. Twenty-one potential participants (14 women, 7 men) met the inclusion criteria. The mean age was 69.2 (± 5.8) years, 38% had a high school education, 24% had a graduate degree, and 66% classified as “at risk for falls”. Eighteen participants agreed they would like to use ST to help improve their balance, and 17 agreed or strongly agreed they would feel confident using the system in either the senior center or the home. Thirteen participants felt confident they could actually set up the system in their home. The mean System Usability Scale (SUS) score was 65.5 ± 21.2 with a range of 32.5 to 97.5. Ten participants scored ST as an above average usability experience compared to other technologies and 5 participants scored a less than optimal experience. Exploratory analysis revealed no significant relationships between user experience, education background, self-described computer experience, and fall risk.

**Conclusions:**

Results support the virtual delivery of the OEP by a Kinect camera and an avatar may be acceptable to older adults from a variety of backgrounds. Virtual technologies, like Stand Tall, could offer an efficient and effective approach to bring evidence-based fall prevention programs to scale to address the problem of falls and fall-related injuries. Next steps include determining if similar or better outcomes are achieved by older adults using the virtual OEP, Stand Tall, compared to the standard of care.

## Introduction

Falls are a tremendous problem facing older adults. It is estimated that 1 in 3 adults age 65 and over fall annually, costing the health care system over US $30 billion dollars in direct medical costs [1]. Older adults fall due to a complex interaction of risk factors [2]. Many factors for falling (ie, age, vision impairment, balance impairment, hearing impairment) increase in risk with increasing age. Therefore older adults, regardless of functional status, are always at some level of risk.

Clinical practice guidelines recommend all older adults be screened annually for fall risk [3]. Older adults who screen positive should have a comprehensive fall risk assessment to determine contributing risk factors. Evidence-based interventions should be prescribed to address all identified factors [4].

One of the most effective interventions to prevent a fall for community-dwelling individuals at low or moderate risk is structured and progressive strength and balance exercises [5,6]. The recommended minimum dose of strength and balance exercise to achieve a protective effect against a fall is 2 hours/week, and the exercise must be ongoing to maintain this protective effect [7-9]. Current fall risk management models must innovate to deliver this dose of exercise without continued medical or physical therapy oversight, which is time and resource-intensive.

The Otago Exercise Program (OEP) is an evidence-based fall prevention program developed in the late 1990s at the University of Otago, New Zealand [10,11]. The OEP has consistently demonstrated a 35% reduction in falls in at risk, community dwelling older adults [12]. The program is designed to be delivered by a physical therapist (PT) in 5 visits over 8 weeks with monthly follow up phone calls, and visits at 6 and 12 months. During the visits, the PT evaluates and prescribes the appropriate strength and balance exercises from the OEP. When the older adult has improved their strength and mobility, they are also prescribed a walking program to complete three times a week. After the initial 8 weeks, the older adult continues to independently complete the prescribed exercises 3 times a week and their walking program 3 times a week for the 12 months of the program [13].

Though highly effective, this program requires either a high level of compliance from the older adult or significant time and resources from the PT. Given the demographics of the world’s aging population and the limited number of PTs, alternative methods to broadly disseminate the OEP must be investigated to have the greatest impact on the problem of falls.

Innovative digital rehabilitation solutions may provide a feasible model to address these challenges. Three-dimensional motion tracking cameras, like the Microsoft Kinect, can guide an older adult through a series of exercises to improve their strength and balance. The technologies have the capability to track the total amount of time spent exercising, the number of repetitions and sets of each exercise, and the quality of the movements performed [14].

Gaming systems for rehabilitation are gaining popularity and acceptance with clinicians and researchers [15]. Several research studies support that exergames, as an adjunct to or in place of traditional rehabilitation for older adults, show promise as an intervention to positively impact functional outcomes [15-17], and can improve balance. [18-20].

Though in early stages, the results from these studies support that exergames may offer a viable option to engage older adults in physical and exercise activity. These results support further exploration of using these systems to deliver proven programs like the OEP to older adults with balance impairments. An important step in this process is to explore the feasibility of using the Kinect to deliver the OEP with a diverse group of older adults. It is unclear from previous studies if older adults from a variety of education and computer experience backgrounds would want to use a virtual system to improve their balance. It is also unknown if older adults with balance impairments or lower levels of functional mobility will be able to or want to independently use these technologies specifically as a fall risk management strategy. Answering these questions will provide insights to the viability of virtual health solutions to bring fall prevention programs to scale.

The purpose of this study was to explore if older adults from a variety of educational backgrounds, self-described computer experience, and level of fall risk could successfully navigate and interact with “Stand Tall” (ST), a virtual translation of the OEP. Stand Tall is the fall prevention exercise program developed using the Virtual Exercise Rehabilitation Assistant (VERA) software. The VERA software uses a Kinect camera and a laptop to deliver therapeutic exercise programs for use independently in the home, rehabilitation or senior center settings.

## Methods

### Participants

All recruitment took place at a senior center in San Diego, California. Participants were recruited via informational flyer. The flyer described the study as a 90-minute session to interact with a virtual technology designed to improve balance. Participantss were compensated US $50 upon completion of the study. Interested participants were connected to the study personnel by the senior center’s site coordinator. All participants were contacted and screened by study personnel. Individuals were included if they: (1) were at least 60 years of age or older, (2) were able to walk independently with or without an assistive device, (3) were able to independently rise from a chair, (4) lived in a noninstitutional setting, (5) self-reported they had at least one chronic disease (arthritis, diabetes, hypertension, etc.), (6) were able to speak, hear, and understand English, and (7) had 20/20 vision from 10 feet with or without glasses.

Participants were excluded if they self-reported that they had been hospitalized in the past 6 months or had a diagnosed cognitive impairment or diagnosed neurodegenerative disease (eg, Parkinson’s disease). The study received IRB approval from Western Institutional Review Board. Twenty-one adults volunteered for the study. All 21 volunteers met the inclusion criteria, signed informed consent prior to the testing session, and completed the 90-minute testing session.

### Data Collection

All data collection took place at the senior center by trained study staff, and included an in-person survey-demographics including highest education obtained, self-reported function, and self-reported computer use. Education is often predictive of adoption of new technologies for older adults [21,22]. Therefore, participants were asked if they had less than 12 years of education (high school or less), an associate’s degree, bachelors, or graduate degree. We developed the following question to gauge how older adults perceived their computer abilities: (1) I don’t use computers, (2) Novice-I know how to turn it on and can use it with help, (3) Average-I can turn it on, launch programs, and email with minimal to no help, and (4) Expert-I use computers without any assistance and can solve most challenges.

**Figure 1:**
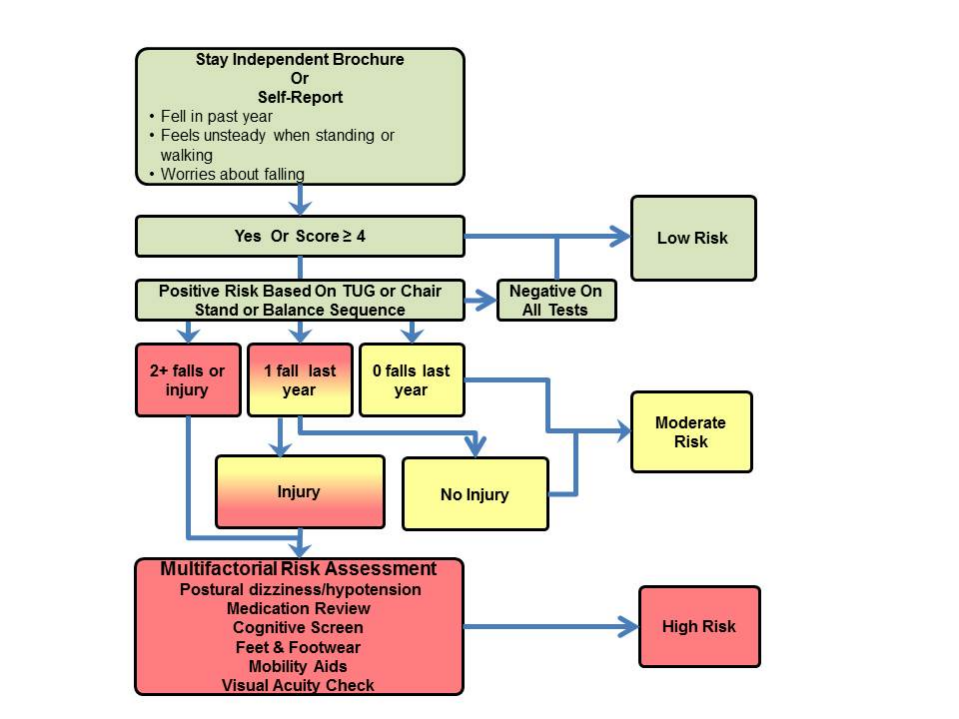
Classification of fall risk based on the Centers for Disease Control's STEADI fall risk assessment tool.

According to the Centers for Disease Control and Prevention (CDC), all older adults are at some level of risk for falling. Quantifying risk is based upon a combination of self-reported risk factors, impairments in strength and balance, history of falls, and additional factors [23]. To standardize fall risk screening, the CDC created the Stopping Elderly Accidents, Deaths & Injuries Fall Risk Algorithm (STEADI) (see Figure 1). The STEADI tool recommends either using the Stay Independent Fall Risk Self-Assessment Tool (Stay Independent) [24] or administering 3 questions to screen for self-reported risk factors. Three physical performance measures, the Timed Up and Go (TUG), the 30-second chair stand (CS), and the four-stage balance test (FS) are the recommended screening tools for impairments in strength and balance.

The Stay Independent was used to screen for self-reported risk factors in this study. The Stay Independent is a 12-item validated tool provides a comprehensive picture of fall risk. Participants who score 4 or more are considered at risk for a fall [24]. Participants completed the TUG, CS, and the FS to assess for impairments in strength or balance [5]. Scoring below established cutoffs for any one of the 3 tests is considered a risk factor for falls. The TUG consists of rising from a chair, walking at the Participant’s usual speed for 3 meters, turning, walking back, and sitting down. Timing starts when the participant rises from the chair and stops when they sit. The cutoff score is 12 or more seconds [25]. The CS consists of the number of times the older adult can rise from a chair in 30 seconds without using their arms. Cutoff scores are based on age and gender-based normative values [26]. The FS is a standing balance sequence which consists of holding a series of progressively more narrow positions for at least 10 seconds. Those who cannot hold the “tandem stance” (heel-toe) position for at least 10 seconds or the single leg stance (standing on one leg) for at least 10 seconds, are considered at risk [27].

Upon completion of baseline data collection, the usability session was started. All usability sessions were videotaped using Morae Usability Software (Techsmith). The video captured both the Stand Tall screen and the individual’s interaction with the technology using a picture-in-picture (PiP) template. Video was started when the participant was introduced to the system and stopped when the participant had exited the room.

Upon completion of the usability study, participants completed a 4-item debrief survey, developed by the study personnel. The survey items were on a 4-point Likert Scale developed specifically to assess participant’s confidence setting up and using the Stand Tall product in the senior center or in the home. Participants completed the standardized and validated System Usability Scale (SUS). The 10-item SUS is a 5-point Likert scale which seeks the subjective opinion about the user experience. Considered “technology agnostic” the SUS has been used in over 3500 studies to determine usability of mobile phones, websites, and software. Scores of > 68 are considered to be an “above average” experience for the user [28,29].

### Testing Procedure

Participants completed baseline questionnaires and functional performance tests. Upon completion of baseline assessments, the video was started and participants were oriented to ST.

Study personnel turned on the system. They explained the participant would see 2 images on the screen, one was an animated avatar named VERA that would demonstrate the exercises, and the other was the participant’s image as a silhouette. Participants were told to listen to VERA’s instructions, observe VERA doing the exercise, and then try the exercise. Study personnel demonstrated calibration, navigation of the application including the “hand swipe” gesture, accessing and completing the exercises, and noted errors, tips and warning messages.

Participants were asked if they were ready to try the system. The participants were first instructed to “think aloud” as they navigated the protocol. Participants stood in front of the camera and identified their silhouette. They were told to extend their hand and identify their hand on the screen. Once the participants identified their hand they were instructed how to navigate to the next screen. Study personnel coached the participants in navigation until they demonstrated they could navigate independently. Participants were instructed to ask for help with navigation and exercise explanation only if they could not successfully complete the tasks after at least one attempt.

Participants independently navigated the program and completed 10 repetitions of 8 of the strength and balance exercises from the OEP: sitting knee extension, standing hip abduction, standing knee flexion, sit to stand from a chair, shallow squats, toe raises, standing on one leg, and tandem stance. Participants were told if they experienced any pain or fatigue they could stop an exercise at any point in time. Study personnel noted if a participant was unable to do a single repetition of an exercise due to either not understanding the exercise, experiencing pain, or a software issue. Participants were supervised the entire session in case of a loss of balance.

Upon completion of the final exercise, participants received notification from VERA that the exercise session was complete. Study personnel began the debrief session. Participants completed the debrief survey, the SUS and answered a series of open-ended questions about the user experience. Participants then received a US $50 gift card and the video was stopped.

### Analyses

All data were analyzed using IBM SPSS Statistics for Windows, Version 22.0. Frequencies and distributions were run for all variables. A Chi Square analysis was performed and nonparametric correlations using a two-tailed Spearman’s Rho test to identify any trends between computer ability, fall risk, and SUS scores. SUS scores were a continuous variable, and ability and fall risk were categorical. Education was recoded to high school or less (0) and college (1). The 4 variables to quantify fall risk were recoded for analysis as follows: Stay Independent was transformed to a categorical variable (0-3 = no risk, 4-12 = risk); the 3 physical performance tests were transformed to categorical variables based on risk (TUG > 12 seconds, CR if < age and gender values, FS if only able to achieve stage 2 or less). The physical performance variables were then collapsed into 1 variable that represented all participants who scored below the cutoff for at least one physical performance test.

For the purposes of this paper, fall risk was operationalized as follows: no risk–score of no risk for the Stay Independent and no risk based on balance and strength tests; low risk–score at risk for the Stay Independent and no risk based on balance and strength tests; Moderate risk–score at risk for Stay Independent and at risk for strength and balance or score at risk for strength and balance; High risk–score at risk for Stay Independent, at risk for strength and balance, and 2 or more falls or 1 fall with injury in the past 12 months.

## Results

### Participants

Descriptive statistics show that a diverse group of older adults participated in this pilot study (Table 1). They ranged in age from 61 to 85, with a mean age of 69.2 (SD 5.8). Of note, there were similar representations of older adults with a high school education or less and those who had completed college. The sample was relatively healthy, with no participant scoring his/her general health as poor. Almost half of the sample (9/21) described themselves as either “very rarely” or “not a user” of technology.

**Table 1:** Characteristics of volunteers (n=21).

General characteristics		Number of participants
**Gender**		
	Female	14
	Male	7
**Race**		
	Black	8
	White (non-Hispanic)	7
	Asian/Pacific Islander	5
	White (Hispanic)	1
**Education**		
	High School/GED	8
	Community college	4
	Bachelor’s degree	4
	Graduate degree	5
**Computer ability**		
	Don’t use	4
	Novice	5
	Average	7
	Expert	5
**Living status**		
	Alone	16
	With spouse	3
	Other	2
**Health status**		
	Excellent/Very Good	12
	Good	6
	Fair/Poor	3
**Fallen in past 12 months**		
	Yes	6
	No	15

All participants were able to complete the 3 physical performance measures to determine if they were at risk. For the TUG, 3 participants took ≥12 seconds, for the CS, 6 participants did fewer repetitions than age and gender-based normative values, and for the FS, 6 participants could only achieve Stage 1 or Stage 2 for at least 10 seconds (Tables 2 and 3).

**Table 2:** Physical performance measures: timed up and go and 30-second chair stand (n=21).

Performance measures		Mean (SD) and range
Timed up and go (measured in seconds)		10.4 (2.5) 6.3–18.5
30-second chair stand (number in 30 seconds)		11.8 (3.8) 6-23

**Table 3:** Physical performance measures –four-stage balance test (n=21).

Stage held for 10 seconds	Highest stage achieved by each participant
Stage 1 (feet side-by-side)	1
Stage 2: semitandem (one foot slightly in front of other)	5
Stage 3: heel-toe position	3
Stage 4: standing on one foot	13

The results of the Stay Independent were summed to identify any participants at risk based on a score of ≥ 4. Per the operational definition, this information was combined with the physical performance measures to identify any participant at low, moderate or high risk of a fall. A total of 11 participants screened positive for fall risk based on self-report. Of those 11, 6 screened negative for strength or balance impairment and were placed in the low risk category and 5 screened positive for a strength or balance impairment and were placed in the moderate risk category. Additionally, 3 participants scored below cut-points for the balance and strength assessment but screened negative for fall risk based on self-report. Finally, 3 of the 8 participants scored below the cutoffs for 2 of the balance and strength measures. Per our definition, participants who scored below cutoffs were placed in the moderate risk category. None of the 21 participants had a combination of history of falls, self-report, and physical performance testing to meet the criteria for “high risk” for a fall (Table 4).

**Table 4:** Fall risk based on self-report and/or physical performance measures (n=21).

No risk	Low risk based on self-report (score of ≥ 4 on Stay Independent Fall Risk Self-Report Tool)^a^	Moderate risk based on balance or strength impairment
7	6	8

^a^ None of the participants had experienced more than 1 fall in past 12 months or a fall with injury.

### Usability Testing

Two participants did not have time to complete the usability questions, and 1 participant’s questionnaire data were not completely captured due to a software malfunction. As a result, for 2 questions we report data on 19 participants and for 2 questions we report data on 18 participants.

The majority of participants (18) either agreed or strongly agreed they would like to use ST to help improve their balance, and 17 agreed or strongly agreed they would feel confident using the system in either the senior center or the home. Over half of participants (13) felt confident they could set up the system in their home.

The SUS scores demonstrated a wide range of user experience. There were 4 participants with missing SUS data. Of the 17 scores, the mean score was 65.5 (SD 21.2) with a range of 32.5 to 97.5. ST was rated as an above average usability experience compared to other technologies by 10 participants. This means they believed the entire experience of using the system, logging on, navigating, pausing, and exercising was at least an acceptable experience. Of these 10 participants, 5 rated the experience at an 80 or above, considered the top 10% of usability experience [30]. However, 5 participants scored a 50 or less, which is a less than optimal experience. Of these participants, 2 interacted with the system when the software crashed multiple times, while the other 3 felt the system had “a ways to go” before they would use it. There were no common characteristics among these 5 participants, they came from a variety of educational and computer experience backgrounds and represented a range of fall risk (Table 5).

Preliminary results from nonparametric correlations suggest that no trends existed between low risk per self-assessment and moderate risk based on physical performance and user experience, education and user experience, or level of computer use and user experience (Table 6).

**Table 5:** Feasibility and interest in using the Virtual Exercise Rehabilitation Assistant.

	Level of Agreement	N
**I would want to use a virtual program to improve my balance (N=19)**		
	Strongly Agree	13
	Agree	5
	Disagree	1
	Strongly Disagree	0
**I feel confident I can set up a virtual program to exercise at home (N=19)**		
	Strongly Agree	6
	Agree	7
	Disagree	3
	Strongly Disagree	3
**I feel confident I can use a virtual program to exercise in my home (N=18)**		
	Strongly Agree	12
	Agree	5
	Disagree	1
	Strongly Disagree	0
**I feel confident I can use a virtual program to exercise at the senior center (N=18)**		
	Strongly Agree	12
	Agree	5
	Disagree	1
	Strongly Disagree	0

## Discussion

### Overview

The purpose of this study was to explore the experience of older adults from a variety of educational backgrounds, computer experience, and level of fall risk navigating and interacting with “Stand Tall”, a virtual translation of the Otago Exercise Program.

Falls in the United States are a universal problem, affecting older adults from all socioeconomic backgrounds. To effectively address the challenges of fall risk management and prevention, it is important to develop a technology that can be adopted by older adults from a wide range of backgrounds and experiences. Given the sample size of 21, we are not able to make definitive statements regarding the relationship between education, level of computer expertise or fall risk and the usability of the system. However, our preliminary analysis for this small group supports that none of these factors had a significant impact on the quality of the experience.

Studies support older adults can and will use exergame-based virtual systems for exercise or rehabilitation [18,31,32]. Researchers have demonstrated healthy older adults can use these systems over extended periods of time and demonstrate significant improvements in function. A group of highly functioning older adults in a retirement community demonstrated they could independently use the Wii Fit for a 6-week balance training intervention and achieved significant improvements in physical performance measures [18]. Similar findings were reported by researchers who developed a series of exergames specifically for older adults. These participants used the system with supervision 2-3 times a week for up to 8 weeks. In addition to significant improvements in function, they demonstrated an adherence rate of 81%, completing their exercise sessions at least four times of five each week [20].

Less is known about older adults’ interest in using a virtual system that is not gamified, but that simply leads the user through a series of balance exercises, much like one would do with a PT. By replicating the experience with a PT we hope to achieve the same outcomes as the OEP when delivered in a traditional model. To our knowledge, this is the first study to explore the feasibility of using a virtual system to deliver a previously validated evidence-based fall prevention program.

The preliminary results support the ST program may be a feasible option to deliver an evidence-based fall prevention exercise intervention to older adults. As a first step to determine if the concept of using technology to bring these programs to scale was feasible, we recruited participants from a broad range of educational, racial, and socioeconomic backgrounds, levels of fall risk, and self-reported experience with technology. To have a representative sample, we developed broad inclusion criteria in which participants self-reported their health status and chronic disease status. The actual chronic diseases for each participant were not documented for this pilot study, unless the participant had a vision or hearing impairment, which would then exclude them from the study. Future work will document the number and types of chronic diseases represented by the study sample, and determine if type of chronic disease has an impact on long-term adherence and compliance with the system.

**Table 6:** Nonparametric correlations for fall risk, computer ability, SUS and education. All Spearman’s Rho correlation coefficients and two-tailed significance levels reported.

		Stay independent	Physical performance	Computer expertise	SUS	Education
**Stay independent**						
	ρ	1.00	.16	.16	.18	.36
	*P*		.49	.50	.49	.11
	N	21	21	21	17	21
**Risk based on TUG or CS or FS**						
	ρ	.16	1.00	.07	.27	-.21
	*P*	.49		.77	.30	.35
	N	21		21	17	21
**Computer expertise**						
	ρ	-.16	.07	1.00	-.10	.28
	*P*	.50	.07		.69	.21
	N	21	21		17	21
**SUS**						
	ρ	-.18	.26	-.10	1.00	-.31
	*P*	.49	.30	.70		.23
	N	17	17	17		17
**Education**						
	ρ	-.36	-.21	.28	-.31	1.00
	*P*	.11	.35	.21	.23	
	N	21	21	21	17	

We did not formally screen any participants for cognitive impairment, but simply asked participants if they had been given a diagnosis of cognitive impairment from their health care provider. Cognitive impairment is a known risk factor for falls, and any fall risk management product needs to take this into account. The goal for this pilot was to have a representative sample from the community interact with the product. The prevalence of mild cognitive impairment (MCI) is approximately 10% for community-dwelling older adults [33]. Individuals with MCI often have slowed reaction times and difficulty inhibiting irrelevant information. It is possible that some of our participants had some degree of MCI which could impact their user experience. Future studies with larger sample sizes will incorporate validated screening tools for MCI to identify participants with cognitive impairment. The goal would be to assess their usability experience and identify key design elements that would create a positive user experience for those with MCI.

There was concern that older adults at either low or moderate risk for falls would either not be interested in using the system, or have a significantly different user experience. The preliminary analysis did not identify any obvious trends to support this assumption. The number of participants for this study is quite small and we anticipate further exploring these trends in future studies.

In the original OEP studies, the average age was 81 and 41% of participants had experienced a fall [11]. The average age of our participants was 10 years younger and 29% (6/21) had experienced a fall. The participants in our study appear to be at a higher level of function compared to the original OEP research. We may find that if we test the system with older adults at a lower level of function that we may see different results. However, 67% of the participants (14/21) were at some level of fall risk or impaired function. This risk level represents older adults who would greatly benefit from a structured strength and balance exercise to prevent a fall; which supports continued exploration of this technology to determine if it would be acceptable for the majority of users.

Our participants represented a range of education levels, socio-economic backgrounds, and functional ability. This diversity of participants has not necessarily been seen in many of the other research reports which have studied a more homogenous population of well educated, highly functioning older adults [18,31,34]. Though preliminary, the finding that 18 participants either agreed or strongly agreed they would like to use a virtual program to improve their balance supports the possibility that ST may be a feasible solution. Previous studies have reported higher levels of technology (internet, text messaging) adoption amongst older adults who were white and with a higher education level [35]. We did not see similar patterns with our users, which may be due to the ease of navigation and using the technology to exercise, as opposed to learning keyboard strokes to seek information. One user with a high school education had never interacted with a computer, nor had she ever participated in a formal exercise class. This participant mastered the technology, was able to complete the 10 exercises, and kept stating, “this is fun, and I need one of these!”

The SUS scores reported for the system demonstrated a wide range of usability experiences. The mean raw score was below that reported in another study that assessed older adult’s usability experience of an exergame [20]. However, the ST program was still in the alpha phase, and the other study assessed a fully functioning system. Given the diversity of usability scores, more testing will be needed on the final product and over a period of time to fully quantify the usability of the system.

Consistent with other studies [15], many participants experienced a feeling of mastery while interacting with the system and statements included, “This is great, when is it coming to the Senior Center?”, “I can do this, it is fun!”, and “I would love to use this in my home, and I see how it would benefit other seniors.” Conversely, when the system did not accurately represent the participant’s efforts, either by over-counting or under-counting repetitions, participants would become frustrated. “What am I doing wrong?” or “Why isn’t she counting me?” were common statements, supporting that a final product must be easy to use but must also be accurate and responsive to provide the optimal user experience.

### Limitations

This was a pilot study and the findings have limited application to the general population. However, our sample represented a broad range of socioeconomic backgrounds, computer experience, and level of fall risk. The lack of any significant relationships between these factors and SUS scores warrants further exploration with larger studies. A second limitation was the results were based on our participant’s interacting with the system one time. We may find that after multiple interactions, participants may gain even more mastery and confidence using the system, and see different trends over time with different participants. A final limitation is, like the OEP, ST is, at this time, an exercise-only intervention. It may be that education and behavior changes, in addition to exercise may be necessary to achieve the best results.

### Conclusion

The results from this study support that virtual delivery of the OEP, by a Kinect camera and an avatar may be a feasible way to scale and disseminate evidence-based fall prevention programs. Older adults enjoy using the technology and value the feedback provided by the avatar on both their form and progress. One of the most effective ways to prevent a fall is to engage in a minimum of 2 hours of strength and balance exercises each week. Virtual technologies like ST could assist older adults in achieving this goal, at a fraction of the resources. Ideally, these technologies would be available in senior centers, YMCAs, and in the home, and would only require a brief orientation to the system and minimal supervision, allowing the older adult to independently engage in an evidence-based program traditionally delivered by a PT. These technologies provide an opportunity for prevention with embedded alert systems that are triggered with changes in performance - either a decrease in weekly adherence or an increase in frequency of errors. Future studies will include determining if older adults can use the program independently over a period of time and determining if older adults who use the ST achieve similar or better outcomes than those participating in a more traditional setting.

## Abbreviations

CDC: Centers for Disease Control and Prevention
CS: 30-second chair stand
FS: four-stage balance test
MCI: mild cognitive impairment
OEP: Otago Exercise Program
PiP: picture-in-picture
PT: physical therapist
ST: Stand Tall
STEADI: Stopping Elderly Accidents, Deaths, and Injury Fall Risk Algorithm
SUS: System Usability Scale
TUG: Timed Up and Go
VERA: Virtual Exercise Rehabilitation Assistant
